# Engaging military couples in marital research: does requesting referrals from service members to recruit their spouses introduce sample bias?

**DOI:** 10.1186/s12874-018-0575-x

**Published:** 2018-10-24

**Authors:** Hope Seib McMaster, Valerie A. Stander, Christianna S. Williams, Kelly A. Woodall, Christopher A. O’Malley, Lauren M. Bauer, Evelyn P. Davila, for the Millennium Cohort Family Study Team, Carlos Carballo, Jackie Pflieger, Cynthia LeardMann, Sabrina Richardson, Evelyn Sun

**Affiliations:** 1 The Henry M. Jackson Foundation for the Advancement of Military Medicine, Inc., 6720A Rockledge Drive, Suite 100, Bethesda, MD 20817 USA; 2 Naval Health Research Center, 140 Sylvester Road, San Diego, CA 92106 USA; 3 Abt Associates, Inc., Central Park West, Suite 210, 5001 South Miami Boulevard, Durham, NC 27703 USA; 4 Leidos, 11955 Freedom Drive, Reston, VA 20190 USA; 5 Emory University, 201 Dowman Drive, Atlanta, GA 30322 USA

**Keywords:** Recruitment, Referral, Dyad, Couples, Military, Epidemiology, Research, Study methodology, Survey, Methods

## Abstract

**Background:**

While enrolling dyads in research studies is not uncommon, there is limited literature on the utility of different recruitment strategies and the resulting selection biases. This paper examined two recruitment strategies used to enroll military couples in a longitudinal study, assessing the impact of both strategies on the representativeness of the final study sample.

**Method:**

Descriptive and bivariate analyses were conducted to 1) identify characteristics associated with spouse referral, 2) compare response rates based on recruitment strategy and assess whether recruitment strategy modified correlates of response propensity among spouses, and 3) assess whether referred spouse characteristics differed from non-referred spouses in the final sample. The study sample consisted of married US service members with 2–5 years of military service as of October 2011 and their spouses.

**Results:**

Service members who referred their spouses to participate in the Millennium Cohort Family Study were more likely to be male, have children, serve in the Army, and have combat deployment experience than those who did not refer their spouse. Nearly two-thirds (*n* = 5331, 64.9%) of referred spouses participated in the Family Study, compared with less than one-third (*n* = 3458, 29.5%) of directly contacted spouses. Spouse characteristics also differed significantly between recruitment groups.

**Conclusions:**

Overall results suggest that minimal bias was introduced by using a referral recruitment methodology. Service members appeared to be more likely to refer their spouses if they perceived the research topic as relevant to their spouse, such that male service members with combat deployment experience were more likely to refer female spouses caring for multiple children. Referred spouses were significantly more likely to respond to the Millennium Cohort Family Study survey than those who were directly contacted; however, the overall success rate of using a referral strategy was less than recruiting spouses through direct contact. Differences between referred spouses and spouses contacted directly mirrored service member referring characteristics.

## Background

Recognizing the importance of human relationships and social interactions, researchers may seek to recruit and study related individuals, such as family members, co-habitants, co-workers, and sexual partners, rather than unrelated individuals. Indeed, many research questions are optimally addressed, or can only be addressed, by using information from dyads. For instance, data from both spouses of a couple are necessary to investigate interpersonal aspects of marriage [1, 2]; other types of family dynamics are most appropriately explored using parent-child or sibling dyads [3, 4]. Medical researchers interested in patient outcomes may study patient-caregiver dyads [5, 6] and often conduct studies of twins in order to tease apart genetic and environmental influences on health [7]. Despite their importance, dyadic research designs present a multitude of methodological complexities [5], beginning with the challenge of recruiting a representative sample of participant pairs.

The recruitment of dyads generally involves many of the same strategies used in traditional subject recruitment, such as placing advertisements in newspapers, approaching patients in hospitals or clinics, posting flyers, or taking advantage of samples of convenience (e.g., students), and some of these may limit the generalizability of findings [1, 8–11]. Furthermore, in dyadic studies researchers must somehow arrange to solicit both members of each potential dyad and ensure in the final dataset that both partners have volunteered together. Unfortunately, many research reports fail to provide valuable details concerning these strategies, and they rarely report rates of nonresponse—for instance, for husbands and wives separately [8, 10], information useful in determining the generalizability of the sample. It is even less common to have some form of public record available for the entire recruitment population (e.g., marriage license records), so the representativeness of the sample can be empirically determined and reported [8].

It is not uncommon to begin the recruitment of a dyad by soliciting the participation of one person and then subsequently enlisting their help in approaching their partner through a referral. However, there is scant literature on the utility of referrals in subject recruitment and the potential selection biases that may result from it. In fact, we have only found a few research reports that detail the process of engaging dyads in any manner, typically where recruitment was particularly difficult or where engaging and retaining the dyad in an educational or treatment program was important [9, 10, 12–14]. Unfortunately, even these studies generally did not address the potential for sample bias introduced through differential recruiting methods, such as spouse referral.

One notable exception was a study of recruitment strategy and decision-making in fertility treatment conducted by Preloran et al. [10]. In this study, a woman’s involvement in recruiting her male partner was related to marital quality and the male partner’s motivations for study participation (e.g., to please their wives vs. to help the researchers). In this context, recruiting couples in less stable relationships was difficult overall, but interestingly, women in unstable relationships appeared more motivated to recruit their male partners alone, rather than allowing the researchers to recruit more directly. By documenting and reporting the various recruitment strategies undertaken to enroll participants, the study team was able to better appreciate how each method introduced bias in their sample of dyads. Though the researchers went through considerable effort to explore potential bias in the recruitment of their sample, they were not able to report details on the male partners that chose not to participate, nor on the women that refused participation, because they relied on a sample of convenience.

The primary aim of this study was to describe two recruitment strategies used to enroll the spouses of services members into a large prospective study of military couples, and to assess the impact of recruitment strategy on the representativeness of the final study sample. The use of two different recruitment strategies provided an opportunity to assess selection bias [15–17] by comparing dyadic data collected *with* versus *without* service member referral. Given this unique opportunity, the study sought to address the following research questions (RQs):

- RQ1: What **service member** characteristics are associated with referring spouses?
- RQ2: Did recruitment strategy impact **spouse** response rates or moderate factors associated with response propensity?
- RQ3: Did recruitment strategy impact final **spouse** representation?

This study was facilitated by the availability of sociodemographic information for all of the military couples in the recruitment population.

## Methods

### Study population and procedures

The Millennium Cohort Family Study (Family Study), an ongoing probability-based cohort study, plans to follow 9872 US active duty and Reserve/National Guard service-affiliated married couples for 21 years [18]. The Family Study provides a unique opportunity to assess the impact of a dyadic recruitment strategy on study sample representation. The Family Study includes couple dyads comprising a military spouse who completed the Family Study questionnaire and a service member who participated in the larger Millennium Cohort Study [19–21]. Dyadic recruitment for the Family Study targeted spouses of Millennium Cohort Study participants with 2–5 years of military service who completed a questionnaire during the 2011–2013 survey cycle (*n* = 28,603 eligible service members). Female and married service members were oversampled to ensure adequate representation in the Family Study.

Newly enrolled married service members were asked to refer their spouses to the Family Study upon completion of the online Millennium Cohort Study questionnaire by providing contact information for their spouse (postal and email addresses.^1^). Approximately one-third of spouses were referred, so the study team was able to invite them to participate immediately using both postal mail and email address information. In cases where contact information was not provided, but the service member did not specifically decline the request to contact his/her spouse, contact information obtained from Department of Defense (DoD) administrative records was used to invite these spouses by postal mail. In order to successfully engage spouses, considerable attention was given to designing each contact (both postal and email) for this study. The theoretical basis for designing each contact drew heavily from social exchange theory as presented by Dillman et al. [22]. The Family Study methods are described in more detail elsewhere [23].

### Analytic subgroups

To investigate our RQs, three analytic subgroups were utilized: (RQ1) newly enrolled **married service members** who completed the 2011–2013 online Millennium Cohort Study questionnaire (*n* = 25,017); (RQ 2) **spouses invited** to complete the Family Study questionnaire either via service member referral or direct contact, excluding spouses of service members who responded by paper where no referral option was offered and spouses with insufficient contact information available from military records (*n* = 19,937); and (RQ3) **spouse responders** to the Family Study questionnaire, excluding spouses of service members who responded by paper (*n* = 8743). For RQ1, service member respondents were categorized into one of two recruitment groups: those who referred their spouse to the Family Study (Referring) and those who either refused to refer their spouse or submitted the survey without responding to the referral item (Refuse/Skip.^2^). For RQ2, spouses were categorized as responders and nonresponders to the Family Study survey. For RQ3, spouse respondents were categorized into two final status groups: those who were referred (Referred) and those who were contacted without a referral (Direct).

## Measures

### Demographic and military data

Sociodemographic and military data for service members were obtained from DoD electronic personnel files maintained by the Defense Manpower Data Center and included service member sex, age, race/ethnicity, service branch, service component, military pay grade, deployment dates, and number of dependents. Service member participants were categorized as deployers if they had at least one deployment in support of Operations Enduring Freedom, Iraqi Freedom, and New Dawn prior to their survey completion date; otherwise, they were classified as nondeployers. Deployers were further classified as deployed with and without combat, based on an affirmative self-report to any of the combat-like experiences on the Millennium Cohort Study questionnaire (e.g., witnessing death, trauma, prisoners of war, or refugees) or on the Post-Deployment Health Assessment (e.g., feeling in danger of being killed, being in direct combat where you discharged a weapon, or encountered dead bodies or people being killed or wounded) [24]. Additionally, service members self-reported education. Spouses self-reported sex, age, race/ethnicity, household income, employment status, prior/current military experience, education, and number of children in household.

### Stress

The Patient Health Questionnaire [25] was used to capture common stressors experienced in the last 4 weeks by both service members and spouses, including: 1) *financial strain* represented by “financial problems or worries”; 2) *relationship stress* represented by “difficulties with husband/wife, partner/lover, or boyfriend/girlfriend”; 3) *caregiver burden* associated with “taking care of children, parents, or other family members”; and 4) *lack of social support* indicated by “having no one to turn to when you have a problem.” Reponses were provided on a 3-point scale (1 = *Not bothered*, 2 = *Bothered a little*, 3 = *Bothered a lot*); however, responses for each item were collapsed to create a binary variable that distinguished those who responded “not bothered” from those who responded “bothered a little or a lot.”

### Stress of military life

Spouses were asked to report the average number of hours per week their service member worked during the past month, as well as the total number of days in the past year the service member was away from home, inclusive of deployments, training, and temporary duty assignments. In addition, spouses completed the Work–Family Conflict Scale [26] modified for military families (5 items from 1 = *Strongly disagree* to 5 = *Strongly agree*; α = .90). Spouses also rated nine military life experiences categorized into Deployment Stress (e.g., combat-related assignment), Injury Stress (e.g., caring for ill, injured, or disabled spouse), and Family Stress (e.g., military duties interfering with family time) based on a 4-point Likert scale ranging from 1 (*Not stressful at all*) to 4 (*Very stressful*), if they had experienced the event in the last 12 months. Events were assigned a score of 0 if spouses reported not experiencing the event in the last 12 months.

### Marital quality and duration

Marital quality was reported by spouses on the Family Study questionnaire using four items from the Quality of Marriage Index [27], which were used previously in large-scale, epidemiological research with deploying military personnel [28]. Items were rated on a 5-point scale (from 1 = *Strongly disagree* to 5 = *Strongly agree*) and averaged to create an overall scale score ranging from 1 to 5. Spouses also self-reported length of marriage.

### Individual health and adjustment

Overall physical and mental health were assessed for service members and spouses using the Medical Outcomes Study Short Form 36-Item Health Survey for Veterans [29, 30], which yields eight scales that combine to create mental component summary (MCS) and physical component summary (PCS) scores. PCS and MCS scores were categorized into three groups (highest 15th percentile, middle 70th percentile, or lowest 15th percentile) to approximate one standard deviation around the mean.

### Statistical analyses

Descriptive statistics and bivariate analyses, including chi-squared tests for categorical variables and *t*-tests for continuous variables, were performed to identify service member and spouse characteristics associated with Family Study recruitment. In order to assess the adjusted association between service member characteristics and referral choice (RQ1), logistic regression was performed. Spouse response rate comparisons (RQ2) were assessed using chi-squared tests. To determine if recruitment group moderated characteristics associated with spousal participation, logistic regression was used to test for interactions with recruitment group (RQ2) in modeling response propensity [18]. Finally, to investigate whether there were significant demographic differences across recruitment groups represented in the final sample (RQ3), a series of chi-squared tests and *t*-tests were performed to examine differences in demographic, military, individual health and adjustment, marital, military life, and family variables. All analyses were weighted to account for nonresponse to the Millennium Cohort Study questionnaire. All analyses were conducted using SAS software, version 9.4 (SAS Institute, Inc., Cary, NC, USA).

## Results

### RQ1: What service member characteristics are associated with referring spouses?

Descriptive characteristics of married service member participants for the overall sample and by referral choice are presented in Table 1. Of the 25,017 married service members who completed the web survey, the majority was male, 34 years old or younger, identified as non-Hispanic White, and had less than a bachelor’s degree. More than half of service members reported having one or more children; that they were not bothered by their significant other, caring for others, financial stress, or having no one to turn to; and that they were in good mental and physical health. With regard to military characteristics, the majority of service members had deployed with combat and was enlisted, in the Army, and active duty.

**Table 1:** Characteristics of married Millennium Cohort Study participants for overall sample and by referral choice

Service member characteristics	Overall		Referring	
	*N* = 25,017		*n* = 8209	
	*n* ^a^	(%)^b^	*n* ^a^	(%)^c^
Demographics				
Sex				
Male	19,272	(86.1)	6626	(34.1)
Female	5745	(13.9)	1583	(26.8)
Age (years)				
17–24	7207	(32.9)	2241	(32.3)
25–34	15,448	(60.4)	5153	(33.4)
35–44	2082	(6.2)	710	(33.6)
> 44	280	(0.6)	105	(37.4)
Race/ethnicity				
White, non-Hispanic	18,540	(69.1)	6393	(35.3)
Black, non-Hispanic	2202	(12.3)	562	(26.1)
Hispanic	2290	(11.0)	655	(28.6)
Other	1985	(7.7)	599	(30.5)
Education				
Less than bachelor’s	18,250	(81.5)	5760	(32.4)
Bachelor’s or higher	6767	(18.5)	2449	(36.0)
Number of children				
0	10,890	(43.6)	3210	(29.7)
1	6966	(27.9)	2314	(33.8)
2	4605	(18.4)	1648	(35.8)
3 or more	2556	(10.1)	1037	(40.8)
Financial problems				
Not bothered	14,390	(54.0)	4185	(29.2)
Bothered a little or a lot	10,353	(46.0)	3969	(37.9)
Military characteristics				
Deployment status				
Nondeployed	8938	(32.5)	2865	(31.9)
Deployed without combat	3362	(12.7)	891	(26.5)
Deployed with combat	12,594	(54.8)	4443	(35.6)
Service branch				
Air Force	7770	(19.0)	2088	(25.4)
Army	10,976	(49.3)	3976	(35.8)
Marine Corps	2181	(14.6)	744	(33.3)
Navy/Coast Guard	4090	(17.2)	1401	(33.5)
Component				
Active duty	19,863	(80.3)	6305	(32.1)
Reserve/National Guard	5154	(19.8)	1904	(37.1)
Pay grade				
Enlisted	20,434	(90.6)	6550	(32.7)
Officer	4583	(9.4)	1659	(36.3)
Individual health and adjustment				
Mental component score				
Lowest 15th percentile	3578	(16.8)	1375	(37.9)
Middle 70th percentile	16,698	(69.2)	5597	(33.7)
Highest 15th percentile	3578	(14.0)	1109	(32.6)
Physical component score				
Lowest 15th percentile	3578	(16.5)	1325	(37.4)
Middle 70th percentile	16,700	(69.5)	5631	(34.1)
Highest 15th percentile	3576	(14.0)	1125	(31.4)
Posttraumatic growth index score, mean (SE)	40.39	(0.08)	40.12	(0.13)
Difficulties with significant other				
Not bothered	16,383	(63.4)	5070	(31.2)
Bothered a little or a lot	8366	(36.6)	3082	(36.8)
Caring for others				
Not bothered	17,188	(68.3)	5210	(30.5)
Bothered a little or a lot	7542	(31.7)	2933	(39.0)
No one to turn to				
Not bothered	20,145	(79.7)	6429	(32.2)
Bothered a little or a lot	4530	(20.4)	1713	(37.6)

Abbreviation: *SE* Standard error

^a^Frequencies are unweighted and may not sum to the total population presented because of missing data

^b^Column percentages and means are weighted and normalized to the study sample

^c^Row percentages and means are weighted and normalized to the study sample

Table 2 presents the logistic regression model examining the relationship of service member demographics, military characteristics, and individual health and adjustment with spouse referral. The unadjusted odds ratio for each variable is also presented to show bivariate contributions to spouse referral. When considered simultaneously in the fully adjusted model, service member demographic characteristics associated with referring spouses to the Family Study were male sex, bachelor’s degree or higher, more than one child, and feeling bothered by financial stress. Service members between 25 and 44 years of age and minorities were *less* likely to refer their spouse. Military characteristics revealed those deployed with combat, and Reservists or National Guardsmen were *more* likely to refer. Additionally, those who reported physical health (PCS) in the highest 15th percentile were significantly *less* likely to refer their spouse to the Family Study. Lastly, service members were *more* likely to refer their spouse if they were bothered by relationship difficulties and by caring for others.

**Table 2:** Unadjusted and adjusted ORs for service member characteristics predicting service member choice to refer (*N* = 23,319)

Service member characteristics	Unadjusted		Adjusted^a^	
	OR	95% CI	AOR	95% CI
Demographics				
Sex				
Male	1.43	(1.33–1.54)	1.36	(1.26–1.48)
Female	1.00^b^		1.00^b^	
Age (years)				
17–24	1.00^b^		1.00^b^	
25–34	1.05	(0.98–1.12)	0.90	(0.84–0.97)
35–44	1.04	(0.93–1.17)	0.71	(0.62–0.82)
> 44	1.25	(0.94–1.67)	0.89	(0.65–1.22)
Race/ethnicity				
White, non-Hispanic	1.00^b^		1.00^b^	
Black, non-Hispanic	0.66	(0.59–0.74)	0.65	(0.58–0.73)
Hispanic	0.76	(0.69–0.85)	0.71	(0.64–0.80)
Other	0.81	(0.73–0.91)	0.83	(0.73–0.93)
Education				
Less than bachelor’s	1.00^b^		1.00^b^	
Bachelor’s or higher	1.14	(1.07–1.23)	1.23	(1.10–1.37)
Number of children				
0	1.00^b^		1.00^b^	
1	1.24	(1.15–1.33)	1.22	(1.13–1.31)
2	1.36	(1.36–1.48)	1.32	(1.21–1.45)
3 or more	1.66	(1.51–1.84)	1.59	(1.43–1.78)
Financial problems				
Not bothered	1.00^b^		1.00^b^	
Bothered a little or a lot	1.50	(1.41–1.59)	1.28	(1.20–1.38)
Military characteristics				
Deployment status				
Nondeployed	1.00^b^		1.00^b^	
Deployed without combat	0.86	(0.78–0.96)	0.97	(0.88–1.08)
Deployed with combat	1.15	(1.08–1.23)	1.10	(1.02–1.18)
Service branch				
Air Force	0.60	(0.56–0.64)	0.70	(0.65–0.76)
Army	1.00^b^		1.00^b^	
Marine Corps	0.89	(0.80–0.99)	0.94	(0.84–1.05)
Navy/Coast Guard	0.89	(0.82–0.97)	1.05	(0.95–1.15)
Component				
Active duty	1.00^b^		1.00^b^	
Reserve/National Guard	1.24	(1.15–1.33)	1.20	(1.10–1.30)
Pay grade				
Enlisted	1.00^b^		1.00^b^	
Officer	1.14	(1.06–1.23)	1.09	(0.97–1.23)
Individual health and adjustment				
Mental component score				
Lowest 15th percentile	1.21	(1.11–1.31)	1.02	(0.92–1.13)
Middle 70th percentile	1.00^b^		1.00^b^	
Highest 15th percentile	0.95	(0.87–1.04)	1.04	(0.95–1.15)
Physical component score				
Lowest 15th percentile	1.15	(1.06–1.25)	1.05	(0.96–1.14)
Middle 70th percentile	1.00^b^		1.00^b^	
Highest 15th percentile	0.88	(0.81–0.96)	0.90	(0.82–0.99)
Posttraumatic growth index score (10 units)	1.00	(0.99–1.00)	1.01	(1.00–1.01)
Difficulties with significant other				
Not bothered	1.00^b^		1.00^b^	
Bothered a little or a lot	1.29	(1.21–1.37)	1.07	(0.99–1.16)
Caring for others				
Not bothered	1.00^b^		1.00^b^	
Bothered a little or a lot	1.47	(1.38–1.57)	1.20	(1.11–1.30)
No one to turn to				
Not bothered	1.00^b^		1.00^b^	
Bothered a little or a lot	1.29	(1.19–1.39)	1.07	(0.97–1.17)

Abbreviations: *AOR* Adjusted odds ratio, *CI* Confidence interval, *OR* Odds ratio

^a^Model adjusted for all covariates listed in the table

^b^Reference category

### RQ2: Did recruitment strategy impact spouse response rates or moderate factors associated with response propensity?

For these analyses, there were 19,937 eligible spouses invited via referral (*n* = 8209) or direct contact (*n* = 11,728) to participate in the Family Study. Nearly half of the invited spouses (*n* = 8789, 44.1%) responded by completing or partially completing the Family Study questionnaire.^3^

Nearly two-thirds (*n* = 5331, 64.9%) of referred spouses responded, compared with less than one-third (*n* = 3458, 29.5%) of those contacted directly (χ^2^ = 2462.72, *p* < .001). To determine the overall success of the referral recruitment strategy, service member referral rate (34%) along with spouse response rate (65%) must be considered. Thus, only 27% of eligible spouses participated using a referral strategy.

To determine if recruitment strategy differentially impacted the likelihood spouses would respond to the survey across demographic subgroups, we tested the interaction of recruitment group (Direct vs. Referred) with 15 service member characteristics (e.g., sex, age, race/ethnicity, children, combat) used to predict response propensity in a previous analysis [18]. Out of all the service member characteristics used to predict Family Study survey response, only the interaction term for children and recruitment group was marginally significant after Bonferroni alpha adjustment (*p* < .003; .05/15). Specifically, having children increased response for spouses contacted directly and slightly decreased response for spouses who were referred (*p* = .003).

### RQ3: Did recruitment strategy impact final spouse representation?

A comparison of Family Study spouse responders across 24 different demographic, military, individual health and adjustment, marital, and family indicators revealed numerous significant differences between recruitment groups (Table 3). Using Bonferroni adjusted alpha levels of .002 per test (.05/24), chi-squared tests and *t*-tests revealed that Referred spouses compared with Direct spouses were more likely to be in the youngest age group (31.7% vs. 25.1%), have 3 or more children (12.4% vs. 8.2%), categorize themselves as homemakers (36.2% vs. 33.5%), and be bothered by financial problems (57.7% vs. 53.3%). With regard to service member military indicators, Referred spouses compared with Direct spouses were more likely to be married to service members with combat deployment experience (59.8% vs. 54.4%), Army affiliation (53.3% vs. 45%), and Reserve/National Guard status (21.9% vs. 18.6%). Additionally, Referred spouses reported service members were away from home due to military duties a greater number of months than Direct spouses (3.6 vs. 3.4). In addition, Referred spouses compared with Direct spouses were more likely to be married less than 2 years (19.4% vs. 14.7%) and report being bothered by difficulties with their significant other (37.2% vs. 32.9%).

**Table 3:** Characteristics of Millennium Cohort Family Study participants by recruitment status (*N* = 8743)

Spouse characteristics	Referred		Direct		*p*-value^c^
	*N* = 5307		*N* = 3436		
	*n* ^a^	(%)^b^	*n* ^a^	(%)^b^	
Demographics					
Sex					.0067
Male	725	(7.4)	401	(6.1)	
Female	4582	(92.6)	3035	(93.9)	
Age (years)					<.0001*
17–24	1339	(31.7)	675	(25.1)	
25–34	3309	(58.8)	2344	(65.7)	
35–44	533	(8.0)	333	(7.5)	
> 44	126	(1.4)	84	(1.6)	
Race/ethnicity					.0285
White, non-Hispanic	4183	(76.5)	2622	(73.6)	
Black, non-Hispanic	226	(5.7)	146	(5.6)	
Hispanic	461	(10.1)	332	(11.2)	
Other	410	(7.8)	315	(9.6)	
Education					.0067
Less than bachelor’s	3198	(67.3)	1979	(64.2)	
Bachelor’s or higher	2097	(32.7)	1447	(35.8)	
Number of children					<.0001*
0	2105	(40.1)	1475	(42.5)	
1	1464	(27.8)	1015	(29.6)	
2	1061	(19.7)	657	(19.7)	
3 or more	677	(12.4)	289	(8.2)	
Employment					<.0001*
Full-time	1730	(29.7)	1284	(35.2)	
Part-time	694	(13.7)	404	(12.1)	
Not employed, looking for work	536	(11.3)	298	(9.6)	
Not employed, not looking for work	246	(4.5)	150	(4.6)	
Homemaker	1849	(36.2)	1130	(33.5)	
Student	237	(4.6)	160	(5.1)	
Financial problems or worries					.0003*
Not bothered	2480	(42.3)	1761	(46.7)	
Bothered a little or a lot	2754	(57.7)	1651	(53.3)	
Military characteristics of the service member					
Deployment status					<.0001*
Nondeployed	1783	(29.9)	1190	(32.2)	
Deployed without combat	594	(10.4)	473	(13.4)	
Deployed with combat	2925	(59.8)	1751	(54.4)	
Service branch					<.0001*
Air Force	1338	(14.4)	1180	(21.8)	
Army	2561	(53.3)	1383	(45.0)	
Marine Corps	485	(14.6)	292	(14.5)	
Navy/Coast Guard	923	(17.8)	581	(18.7)	
Component					.0006*
Active duty	4109	(78.1)	2773	(81.4)	
Reserve/National Guard	1198	(21.9)	663	(18.6)	
Pay grade					.0785
Enlisted	4002	(87.2)	2552	(86.1)	
Officer	1305	(12.8)	884	(13.9)	
Spouse’s military experience					.2629
Never	4374	(85.9)	2784	(84.6)	
Prior	488	(6.7)	341	(7.3)	
Current	432	(7.4)	302	(8.1)	
Individual health and adjustment					
Mental component score					.0028
Lowest 15th percentile	833	(17.7)	462	(14.8)	
Middle 70th percentile	3609	(69.0)	2428	(70.2)	
Highest 15th percentile	772	(13.3)	523	(15.0)	
Physical component score					.0534
Lowest 15th percentile	815	(16.6)	480	(14.8)	
Middle 70th percentile	3640	(69.0)	2391	(69.6)	
Highest 15th percentile	759	(14.3)	542	(15.7)	
Marital					
Years of marriage					<.0001*
< 2	810	(19.4)	380	(14.7)	
2–5	2978	(57.8)	1974	(57.8)	
6–10	1095	(18.5)	826	(22.3)	
≥ 11	338	(4.4)	238	(5.2)	
Difficulties with significant other					.0003*
Not bothered	3414	(62.8)	2366	(67.1)	
Bothered a little or a lot	1806	(37.2)	1048	(32.9)	
Abbreviated quality of marriage index, mean (SE)	17.15	(0.06)	17.13	(0.08)	.8263
Military life					
Months away from home (service member), mean (SE)	3.31	(0.06)	3.02	(0.07)	.0009^*^
Hours of work per week (service member), mean (SE)	45.77	(0.32)	46.40	(0.39)	.2116
Work-family conflict scale, mean (SE)	21.44	(0.08)	21.41	(0.10)	.8255
Deployment stress, mean (SE)	1.67	(0.02)	1.59	(0.02)	.0026
Injury stress, mean (SE)	0.63	(0.02)	0.61	(0.02)	.3100
Family stress, mean (SE)	1.69	(0.02)	1.63	(0.02)	.0417
Family					
Caring for others					.0028
Not bothered	2867	(54.3)	1999	(58.0)	
Bothered a little or a lot	2358	(45.7)	1414	(42.0)	

Abbreviation: *SE* Standard error

^a^Frequencies were unweighted and may not sum to the total population presented because of missing data

^b^Column percentages and means were weighted and normalized to the study sample. Percentages may not sum to 100 because of rounding

^c^*p*-values were calculated with chi-squared tests and t-tests, respectively. They were weighted and normalized to the study population. Using the Bonferroni adjustment, significance was assessed with α = .002

*Values are significant

## Discussion

Few large scale studies have recruited married couples, and among those studies that have, it is unclear whether or how spousal referrals were incorporated into their recruitment strategy [8–11], making it impossible to determine selection bias and representativeness of the study sample. The Family Study offers a unique opportunity to thoroughly examine the potential for bias when one member of a dyad is asked to refer the other by analyzing extensive data collected from military spouse dyads, including sociodemographic and psychosocial characteristics. For this study, we were able to: 1) examine service member characteristics associated with providing a referral compared with those who chose not to refer; 2) determine if spouse survey response was impacted by service member referral choice; and 3) assess whether responding spouses who were referred differed significantly from spouses who were contacted without a referral. To our knowledge, this is the first large scale study to provide a detailed assessment of the impact of a referral recruitment strategy compared with a direct contact recruitment strategy on the representativeness of the final dyadic study sample.

The results of our initial unadjusted analyses examining service member characteristics associated with providing a spouse referral (RQ1) showed that the majority of service member sociodemographic and military variables were significantly associated with referring spouses, along with various mental and physical health composite measures, and financial and social concerns. Similar to the Family Study nonresponse findings [18] and those of other survey research studies, providing a referral is related to sociodemographic characteristics strongly related to participating in survey research. In adjusted analyses, service member sex, age, race/ethnicity, education, and financial concerns were associated with service member referral. Indeed, the strongest predictor of referring one’s spouse to the Family Study was being male followed by having children, with a greater number of children increasing this association. In addition, we found several military factors to be predictive of referral choice, such that those who experienced a combat deployment were more likely to refer their spouse to the Family Study than those who never deployed or deployed without combat; service members in the Reserve/National Guard were more likely to refer than active duty service members; and those serving in the Air Force were the least likely to refer their spouses. Other significant predictors of referral choice were being bothered by caring for others and marital difficulties.

These differences, however, are quite small and likely rooted both in the typical demographic correlates of survey response (e.g., sex and socioeconomic level) and unique motivations to support the objectives of this study (e.g., concerns regarding the impact of military life stress on families). If spousal referrals are similar to physician referrals, then referring spouses may have a greater interest in the research topic than non-referring spouses [31, 32]. That is, the systematic differences associated with service member referral choice seem to reflect a perception of spouse “fit” with the research topic, such that male service members with children, who had experienced a combat deployment, and who may be having difficulties at home see themselves and their spouses as suffering from an “ailment” related to the research topic [33–35]. Female service members may not think their male spouses “fit” with the research topic, especially if the spouse is also a service member or they do not have children.

Our examination of spouse response by referral status (RQ2) revealed that referred spouses were significantly more likely to respond to the Family Study survey than those who were not referred. This difference was likely the partial result of two known methodological differences in recruitment strategy. Spouses who were referred to the study with complete contact information were invited to participate in the Family Study immediately by email and within 2 weeks by postal mail, whereas there was a delay for spouses contacted without a referral. In addition, Referred spouses were contacted multiple times by email to complete the questionnaire and were provided an email embedded link to the web survey, whereas Direct spouses were only invited by postal mail to complete the web survey.^4^

Email augmentation has been shown to ease the response task and garner greater attention to the response request, resulting in improved response rates [22].

In addition to the methodological differences, our results indicate that service members are more likely to refer spouses who may have an interest in the study topic based on their perception of spouse “fit” with a survey of military families. That is, service members may have been more likely to refer their spouses if they thought their spouse would respond. Indeed, spouses may share this perception of survey fit—increasing the likelihood of interest in the study topic—and of survey response [22].

Although Referred spouses were significantly more likely to respond than Direct spouses, the success rate of using a referral strategy was less than when recruiting spouses directly. That is, the direct recruitment method provided a much greater number of spouses to be contacted than the referral recruitment method, so even though the response rate was lower for the Direct group than the Referred group, the percentage of eligible spouses responding using the Direct method was higher than when relying on referral. In other words, inviting spouses without a referral was more successful than inviting *only* spouses who were referred.

Lastly, we used a nonresponse model developed for the entire Family Study population to look for interactions between 15 response propensity characteristics and recruitment group on spouse survey response. We found that for all but one interaction, recruitment group did not significantly modify the relationship between nonresponse characteristics of the spouses invited to the Family Study and survey response. Only the interaction term for children and recruitment group was marginally significant, suggesting that having children slightly decreased the likelihood of response for the Referred group and slightly increased the likelihood of response for the Direct group. This suggests that recruitment strategy, for the most part, had little impact on spouse response characteristics.

A final comparison of Family Study participants (RQ3) revealed numerous differences between recruitment groups that closely mirrored the service member characteristics associated with referring spouses. That is, Referred spouses compared with Direct spouses were younger, had children, and reported being bothered by financial problems, marital problems, and caring for others. Likewise, the military characteristics of Referred spouses reflected those of Referring service members (e.g., combat deployment, Army, Reserve/National Guard). In addition, Referred spouses were more likely to describe their employment status as homemaker, to be married less than 2 years, and to report their service member spent more months away from home compared with Direct spouses. Again, these differences appear to reflect perceptions of “fit” with the research topic. We suspect these characteristics may indicate more identification with the stereotypes for military spouses, as well as greater interest in the focus and objectives of the study as a motivation for referral and participation. Furthermore, this also indicates that the total picture of military operational stress, rather than the difficulties of deployments alone, are perceived as relevant and should continue to be a focus in the Family Study as well as other research on military families.

## Conclusion

Overall results suggest that minimal bias was introduced into the Family Study by using a referral recruitment methodology. Systematic differences in group membership appeared to be driven by service members referring spouses based on perceptions of “fit” with the research topic, which were further augmented by the increased response rate of spouses mirroring those characteristics. These group differences accounted for only a small proportion of variability in final group membership and provide confidence that minimal bias was introduced by using referral recruitment.

## Abbreviations

DoD: Department of Defense
Family Study: Millennium Cohort Family Study
MCS: Mental component summary
PCS: Physical component summary
RQ: Research question

## Footnotes

1 The paper version of the questionnaire did not include a referral request; therefore, paper completers and their spouses were not included in this study.

2 Chi-squared analyses comparing service members that refused to refer (Refuse) with service members that submitted the survey without providing a referral (Skip) revealed no meaningfully significant differences; therefore, these groups were combined.

3 Family Study overall response rates differ from those reported in this study because of exclusion criteria [18].

4 A small portion of Direct spouses (*n* = 2463) were part of an experiment where they were invited to complete a paper version of the survey without being informed of the web option. Results from this experiment indicated that military spouses had higher response rates when asked by postal mail to complete the web survey than when asked by postal mail to complete a paper survey [23], counter to previous research suggesting that changing modes decreases response rates [22].
